# The regional sequestration of heterochromatin structural proteins is critical to form and maintain silent chromatin

**DOI:** 10.1186/s13072-022-00435-w

**Published:** 2022-01-31

**Authors:** Junsoo Oh, Soojin Yeom, Jiyeon Park, Jung-Shin Lee

**Affiliations:** Department of Molecular Bioscience, College of Biomedical Science, Kangwon National University, 1 Kangwondeahak-gil, Chuncheon, 24341 Republic of Korea

**Keywords:** *Saccharomyces cerevisiae*, *Schizosaccharomyces pombe*, Heterochromatin structural proteins, SIR complex, Swi6

## Abstract

**Abstract:**

Budding yeast *Saccharomyces cerevisiae* and fission yeast *Schizosaccharomyces pombe* are good models for heterochromatin study. In *S. pombe*, H3K9 methylation and Swi6, an ortholog of mammalian HP1, lead to heterochromatin formation. However, *S. cerevisiae* does not have known epigenetic silencing markers and instead has Sir proteins to regulate silent chromatin formation. Although *S. cerevisiae* and *S. pombe* form and maintain heterochromatin via mechanisms that appear to be fundamentally different, they share important common features in the heterochromatin structural proteins. Heterochromatin loci are localized at the nuclear periphery by binding to perinuclear membrane proteins, thereby producing distinct heterochromatin foci, which sequester heterochromatin structural proteins. In this review, we discuss the nuclear peripheral anchoring of heterochromatin foci and its functional relevance to heterochromatin formation and maintenance.

**Graphical Abstract:**

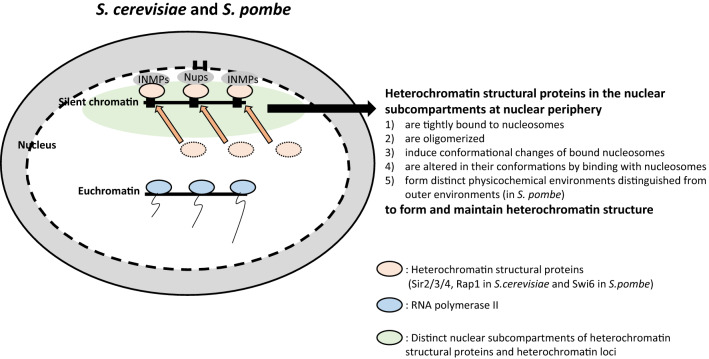

## Introduction

Many DNA-templated processes, such as replication, transcription, and DNA repair, are regulated in the context of chromatin structure, which is classified into euchromatin and heterochromatin. Euchromatin is less condensed and more easily accessed by RNA polymerase II and transcription factors, which enables active transcription. In contrast, heterochromatin maintains highly condensed chromatin regions throughout the cell cycle, impeding the access of various transcription factors and causing gene silencing [1, 2]. In higher eukaryotes, heterochromatin regions are characterized by specific histone modifications, namely the methylation of histone H3K9 and histone H3K27 [1, 3]. H3K9-methylated chromatins are bound by heterochromatin protein 1 (HP1), and this process leads to heterochromatin formation [1, 2, 4, 5].

*Saccharomyces cerevisiae* and *Schizosaccharomyces pombe* are well-studied model systems for the investigation of heterochromatin. However, between the two model species, there are many differences in the mechanism of heterochromatin formation and gene silencing. Notably, although the methylation of histone H3K9 and HP1 are well-conserved in *S. pombe*, neither of this histone modification and heterochromatin factor exist in *S. cerevisiae* [1, 2, 6]. Instead, in *S. cerevisiae*, the formation and maintenance of heterochromatins are regulated by the silent information regulator (SIR) complex-silencing system [2, 6, 7].

Although the mechanisms for silent chromatin formation are different in both model systems, they share several essential common features. Heterochromatin regions and heterochromatin structural proteins, the Sir2/3/4 complex in *S. cerevisiae* and Swi6 in *S. pombe*, are sequestered in several foci at the nuclear periphery [7–11]. After a brief introduction on the mechanism of heterochromatin formation, we will introduce the regional sequestration of both heterochromatin structural proteins and heterochromatin loci at the nuclear periphery [7–11]. We will further discuss how the sequestered nuclear subcompartments contribute to heterochromatin structure and gene silencing. In sequestered nuclear subcompartments, heterochromatin structural proteins and heterochromatin loci form and maintain heterochromatin structure by the following strategies: (1) heterochromatin structural proteins are oligomerized [7, 12–14]; (2) physical interaction between heterochromatin structural proteins and nucleosomes induces conformational changes of each other [7, 12–14]; (3) although not identified in *S. cerevisiae*, H3K9-methylated nucleosomes and Swi6 in *S. pombe* form phase-separated liquid condensates, which maintain distinct biochemical conditions distinguished from the outer environments [7]. Through these strategies, heterochromatin structural proteins are tightly bound to nucleosomes [13, 15]. In addition, neighboring nucleosomes are tightly linked, which enables more compacted chromatin structures and gene silencing [7, 15, 16]. Through this review, we propose the importance of regional sequestration of heterochromatin structural proteins for the formation and maintenance of heterochromatin structure by exemplifying two distinct models, *S. cerevisiae* and *S. pombe.*

### Heterochromatin structural proteins are recruited to heterochromatin loci via different mechanisms in *S. cerevisiae* and *S. pombe*

In *S. cerevisiae*, epigenetic silencing markers conserved in metazoans have not been identified. Specifically, there are no orthologous proteins of chromatin modifiers for silencing mechanisms, such as DNA cytosine methylation, H3K9 methylation, and H3K27 methylation, in *S. cerevisiae*. Instead, the silent chromatin structure in *S. cerevisia*e is maintained through the recruitment and spreading of the SIR complex, which is composed of Sir2, Sir3, and Sir4 [6].

In *S. pombe*, H3K9 methylation and Swi6 lead to heterochromatin formation. H3K9 methylation is a conserved histone modification, responsible for heterochromatin formation in a multitude of organisms from fission yeast to humans [3]. Clr4 is the only H3K9 methyltransferase in *S. pombe*, but several H3K9 methyltransferases have been identified in mammalian cells, including SUV39H1/KMT1A, SUV39H2/KMT1B, SETDB1/KMT1E, dimeric `G9a/KMT1C-GLP (G9a-like protein)/KMT1D, and the PRDM family [17]. *Drosophila melanogaster* retains Su(var)3–9, G9a and SETDB1 as the H3K9 methyltransferases [18]. In *Arabidopsis thaliana*, KYP, SUVH5, and SUVH6 are identified as H3K9 methyltransferases [19]. In *Neurospora crassa*, a filamentous fungus, the H3K9 methyltransferase is Dim5 [20].

In *S. cerevisiae*, the SIR complex is recruited to silent loci by interaction with repressor proteins bound to heterochromatin-specific DNA elements, including silencers in silent mating loci and multiple Rap1 binding sites in telomeres [6]. In *S. pombe*, Swi6 is recruited to silent loci by binding to H3K9-methylated nucleosomes [1, 4, 21]. Upon recruitment, both heterochromatin structural proteins are spread from nucleation sites through self-assembly [1, 6, 22].

### Recruitment of SIR proteins in silent chromatin regions is regulated by DNA elements and cognate binding proteins

**Fig. 1:**
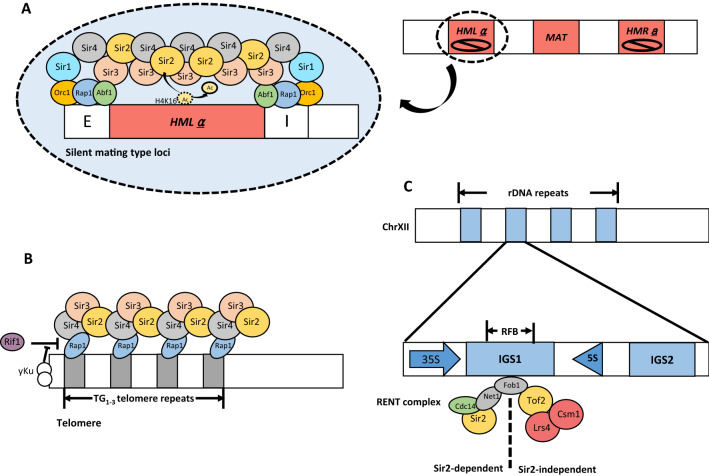
Heterochromatin formation in *Saccharomyces cerevisiae*. **A** Heterochromatin formation in silent mating-type loci of *S. cerevisiae*. At budding yeast chromosome III, two silent mating-type loci—*HML*α and *HMR*a—surround the mating-type (*MAT*) locus. Each homothallic mating (*HM*) locus is surrounded by two proto-silencers, *E* and *I*, which nucleate the heterochromatin assembly. Silencer elements are bound by Orc1, Rap1, and Abf1. Orc1 interacts with Sir1 and Abf1 interacts with Sir3. Through the self-reinforcing mechanism of the SIR complex composed of Sir2, Sir3, and Sir4, silent chromatin is formed at *HM* loci. **B** Heterochromatin formation in telomeres of *S. cerevisiae.* Telomeres consist of TG_1-3_ repeat regions and chromosomal ends. Chromosomal ends are bound by yKu70/80 heterodimeric complexes. Telomeric repeats contain multiple Rap1 binding sites and the SIR complex is recruited to telomeric repeats through Rap1. Rif1 competes with Sir4 for binding to Rap1. The yKu complex regulates this competition process for Sir4 recruitment and SIR complex assembly. **C** Heterochromatin formation in rDNA repeats of *S. cerevisiae.* Approximately 100 to 200 rDNA repeats are positioned at chromosome XII. Each repeat consists of the 35S pre-rRNA gene and 5S rRNA gene which are separated by intergenic spacer 1(IGS1). IGS2 is located upstream of the 5S rRNA gene. A replication fork barrier (RFB) site is positioned within IGS1 and the binding site for Fob1. The binding of Fob1 into RFB sites causes recombination of rDNA repeats, which should be prevented by the binding of additional proteins. Net1 tethers to Fob1 and recruits Cdc14 and Sir2 into rDNA loci, thereby forming the regulator of nucleolar silencing and telophase exit (RENT) complex. Tof2 binds to Fob1, leading to the recruitment of two cohibin complex components, Lrs4 and Csm1, for Sir2-independent rDNA silencing. Lrs4/Csm1 interacts with Heh1/Nur1 (two nuclear membrane proteins)

In *S. cerevisiae*, homothallic mating (*HM*) loci, telomeres, and rDNA loci are the silent chromatin regions [6] (Fig. 1). The mating type of budding yeast is determined by a gene positioned at the *MAT* locus—either “a” or “α” (alpha) [6] (Fig. 1A). A budding yeast cell, regardless of its mating type, contains all genes for both mating types in two *HM* loci; these *HM* loci—*HMR*a and *HML*α—are located at either side of the *MAT* locus and maintained in a silent state, called *HM* silencing (Fig. 1A) [6]. Telomeres are composed of TG_1-3_ repeats of 300–350 bps in length and cognate binding proteins within the chromosomal ends [6] (Fig. 1B). rDNA loci are composed of approximately 100–200 rDNA repeats (Fig. 1C). Each of the repeat includes genes coding 35S pre-rRNA transcribed by RNA polymerase I and genes coding 5S rRNA transcribed by RNA polymerase III; these regions are separated by intergenic spacer (IGS) regions containing IGS1 and IGS2 (Fig. 1C) [23, 24].

Specific DNA elements in *HM* loci, telomeres, and rDNA loci are bound by cognate binding proteins, which recruit SIR proteins to silent chromatin regions. Silent *HM* loci are positioned between two silencers, *E* and *I*, and silencers are bound by repressor and activator protein 1 (Rap1), autonomously replicating sequences (ARS) binding factor 1 (Abf1), and origin replication complex (ORC) [25, 26] (Fig. 1A). These silencer-binding proteins form a loop structure and recruit the SIR complex to nucleate silent chromatin formation [26–28]. For example, Abf1 binds to Sir3, and ORC interacts with Sir1, thereby bringing Sir4 into silent loci [6, 29, 30]. After being recruited to chromatin via interaction with Sir4, Sir2, which is a protein of the NAD^+^-dependent histone deacetylase (HDAC) family, deacetylates acetylated H4K16 (H4K16ac) [31–34]. Deacetylation of acetylated H4K16 enhances the access of Sir3 to chromatin and blocks H3K79 methylation [31–34]. In telomeres, Rap1 and the yKu70/80 heterodimeric complex are bound to multiple Rap1 binding sites in TG1-3 repeats and chromosomal ends, respectively [35]. The Sir3–Sir4 dimer is recruited to subtelomere regions by binding to the carboxy-terminal domain of Rap1 [36, 37]. The interaction between Rap1 and Sir4 is inhibited by Rif1, and the yKu70/80 heterodimeric complex contributes to the binding of Sir4 to Rap1 by suppressing the inhibitory effect of Rif1 [35]. In rDNA loci, the mechanism of silent chromatin formation at the IGS1 region has been studied more than that at IGS2 (Fig. 1C). The replication fork barrier (RFB) site is positioned within IGS1 and bound by Fob1 [23]. Net1 is bound to Fob1 and brings Cdc14 and Sir2 to form the regulator of nucleolar silencing and telophase exit (RENT) complex [38, 39]. Topoisomerase associated factor 2 (Tof2) is bound to Fob1 and brings two cohibin complex components (Lrs4 and Csm1) [23, 40, 41]. Lrs4 and Csm1 directly recruit the condensin complex and enable correct alignment between sister chromatids [42, 43]. Therefore, unequal sister chromatid exchange is prevented, and the stability of rDNA regions is increased by the condensin complex [42].

### In *S. pombe*, H3K9 methylation domains are formed at heterochromatin regions to initiate Swi6 recruitment

**Fig. 2:**
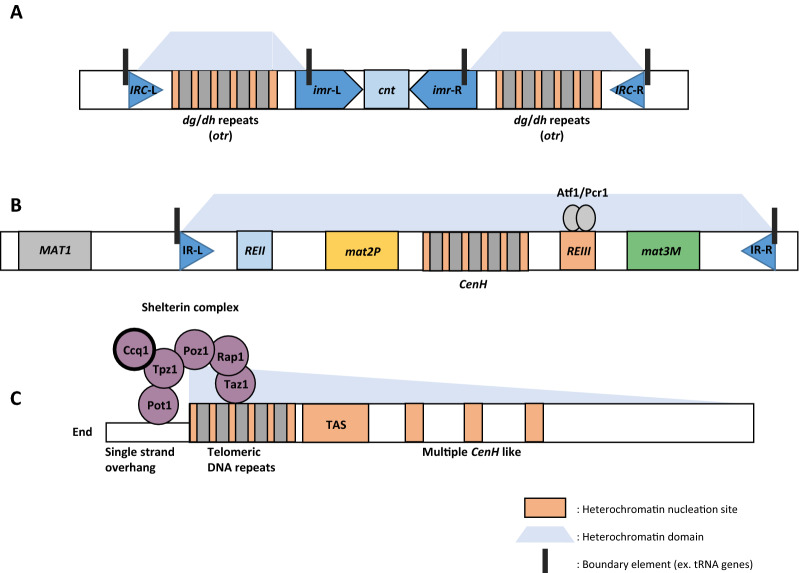
Heterochromatin formation in *Schizosaccharomyces pombe*. **A** Heterochromatin formation in centromeres. In centromeres, centromeric repeats (*cnt*) are surrounded by two innermost repeats (*imr*) regions with inverted orientation. Outer repeat (*otr*) elements are positioned further outside, and they consist of *dg*/*dh* repeat elements. *dg/dh* repeats nucleate heterochromatin in an RNAi-dependent manner. Once nucleated, heterochromatin is spread through the H3K9me-Swi6-dependent self-reinforcing mechanism up to its encounter with boundary elements including tRNA gene clusters. **B** Heterochromatin formation in silent mating-type loci. The mating-type is determined by the *MAT1* gene; two mating-type determining regions, *mat2*P and *mat3*M, which contain information on both mating types, are maintained in silent chromatin. *CenH* regions show high homology to centromeric *dg/dh* repeats and function as RNAi-dependent nucleation centers. The *REIII* element is the binding site for Atf1/Pcr1, constituting an RNAi-independent heterochromatin nucleation site. Once nucleated, heterochromatin is spread up to its encounter with boundary elements, IR (inverted repeats)-L and IR-R. **C** Heterochromatin formation in telomeres. Telomeres are composed of double-stranded telomeric DNA repeats and single-stranded overhangs at chromosomal ends. Telomeric DNA repeats and immediately adjacent telomere-associated sequences (TAS) are RNAi-independent heterochromatin nucleation sites. Regions more distal to chromosomal ends contain multiple *CenH*-like sequences, functioning as RNAi-dependent heterochromatin nucleation sites (e.g., centromeres and silent mating-type loci). The shelterin complex consists of Tpz1/Pot1 subcomplexes bound to single-stranded overhangs and the double-stranded telomeric repeat-binding protein Taz1 connected by Rap1 and Poz1. The shelterin component Ccq1 recruits Clr4 for H3K9 methylation and subsequent Swi6-dependent heterochromatin formation. Additionally, Ccq1 leads to SHREC recruitment for transcriptional gene silencing. There is no known boundary element at telomeres; therefore, telomeres contain a long transition zone showing a gradual decrease in the heterochromatin domain

H3K9 methylation domains in heterochromatin regions are important for the chromatin recruitment of Swi6 and are initiated in heterochromatin nucleation sites [4, 21]. The representative heterochromatic regions in *S. pombe* are pericentromeric regions as well as telomeres and silent mating-type loci (Fig. 2). *S. pombe* possesses 10-kb centromeric regions, which are large compared to the much smaller 125-bps centromeres in *S. cerevisiae* (Fig. 2A) [44]. The central region of the centromeres is composed of two classes of DNA sequences, *imr* (innermost repeat) and *cnt* (central) repeats. The *cnt* region is flanked by two *imr* sequences in an inverted orientation [1, 45]. This central region, consisting of *cnt* and two *imr* sequences, is flanked by *otr* (outer repeat) repeats [46]. This pericentromeric *otr* repeats contain *dg* and *dh* repeat sequences, which are the RNAi-dependent heterochromatin initiation sites [46]. The mating type of fission yeast, either + or −, is determined according to the gene—either *mat2P* or *mat3M*—located at the *MAT* locus. Like *S. cerevisiae*, both *mat2P* and *mat3M* elements are maintained in a transcriptionally silent state (Fig. 2B) [47, 48]. Two heterochromatin initiation sites, *CenH* region and *REIII* elements, are located between *mat2P* and *mat3M.* The *CenH* region is composed of multiple repeats homologous to centromeric *dg/dh* repeats [45]. In telomeres, chromosomal ends contain approximately 300 bps of telomeric double-stranded DNA repeats, with a single-stranded overhang that protrudes from the ends (Fig. 2C) [49]. Telomere-associated sequences (TAS) refer to the DNA regions proximal to telomeric DNA repeats [49–51]. In addition, multiple *CenH*-like regions were identified in the more distal regions to telomeric DNA repeats [45, 50].

In centromeres, the recruitment of Clr4 and histone deacetylases (HDACs) into centromeric *dg* and *dh* repeats to initiate heterochromatin formation is mediated by RNAi-dependent and -independent mechanisms [52–55] (Fig. 2A). In silent mating loci, RNAi-dependent heterochromatin assembly starts at the *CenH* region, which is homologous to centromeric *dg*/*dh* repeats, and RNAi-independent heterochromatin assembly is initiated at *REIII* elements by the cognate binding proteins Atf1/Pcr1 [1, 56]. Similar to the Atf1/Pcr1-dependent heterochromatin assembly in silent mating-type loci, telomeres form and maintain heterochromatin through the shelterin complex (Fig. 2C) [49, 51, 53, 57]. The shelterin complex is composed of Tpz1, Pot1, Taz1, Rap1, Poz1, and Ccq1 [58–60]. Ccq1 recruits the Clr4 complex (CLRC) for efficient H3K9 methylation and chromatin recruitment of Swi6 [60]. Multiple *CenH*-like sequences function as RNAi-dependent heterochromatin nucleation sites similar to centromeric *dg/dh* repeats [50].

Heterochromatin structure formation is dependent on the spreading of Clr4 and Swi6 [1, 13, 22, 45, 52, 61]. Clr4 contains both chromodomain (CD) and SET domain. Therefore, after binding to methylated H3K9 via CD, it methylates H3K9 at adjacent unmodified nucleosomes, thereby spreading the H3K9 methylation domain [62]. In a Swi6-deletion mutant, however, H3K9 methylation was initiated at the nucleation site, but heterochromatin was not spread enough to cover the entire silent loci [22, 45]. There are two Heterochromatin Protein 1 (HP1) homologs in *S. pombe*, Chp2 and Swi6, which are composed of an amino-terminal CD and a chromo-shadow domain (CSD) separated by a hinge region [63]. These HP1 proteins form homodimers through the CSD–CSD interface [13, 64]. However, even though Chp2 and Swi6 are HP1 homologs, their roles in heterochromatin formation are different; Chp2 is present in low abundance, and it leads to the recruitment of the Snf2/HDAC repressive complex (SHREC), thereby deacetylating H3 lysine14 (H3K14) residue and participating in transcriptional gene silencing by limiting the access of RNA polymerase II at heterochromatin loci [65–67]. On the contrary, Swi6 is highly abundant, and it functions as a heterochromatin structural protein in a dose-dependent manner [65].

Boundary elements sequester the H3K9 methylation domain within heterochromatin sites. In centromeres, the centromere-proximal boundary is the tRNA gene cluster, and the centromere-distal boundary is the inverted repeat flanking left/right sides of centromeres (*IRC-*L/R) regions that retain tRNA genes (Fig. 2A) [1, 45, 68]. However, the *IRC*-R of centromere I does not contain the tRNA gene, but the *IRC* also functions as a boundary [45]. In silent mating loci, inverted repeat (IR) regions and tRNA gene clusters within IR regions form heterochromatin boundaries in the similar manner as centromeres, thus restricting the propagation of the silent chromatin region (Fig. 2B) [45, 69, 70]. Contrary to silent mating-type loci, known boundary elements do not exist at the telomeres; it instead contains a long transition zone showing a gradual decrease in the heterochromatin domain (Fig. 2C) [45, 53].

### Heterochromatin structural proteins are sequestered at the nuclear periphery

In *S. cerevisiae,* there are 32 telomeres from 16 chromosomes. Notably, a previous study reported that SIR complexes were observed under a microscope, with 3–5 clustered foci at the nuclear periphery of each cell, which suggests that distinct silent chromatin regions, including each of the 32 telomeres and *HM* loci, are clustered and form distinct nuclear subcompartments with high SIR complex concentrations [8, 71]. In the same manner, Swi6 is detected as several foci by microscopic analysis in each cell of *S. pombe* [7].

**Fig. 3:**
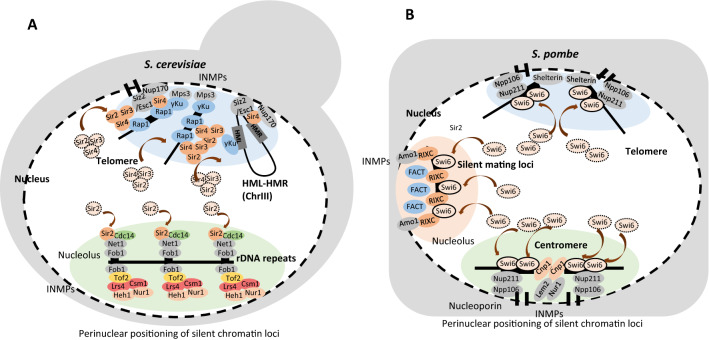
Regional sequestration of heterochromatin structural proteins at nuclear periphery. **A** Perinuclear anchoring of heterochromatin structural proteins in *S. cerevisiae*. Budding yeast silent chromatin loci, such as telomeres, *HM* loci and rDNA repeats are anchored to the nuclear periphery through interaction with inner nuclear membrane proteins (INMPs) and nucleoporins. At rDNA repeats, Net1 sequesters Sir2 and Cdc14 specifically during the S-phase for RENT complex formation. rDNA repeats are anchored to the nuclear periphery through interaction between INMPs Heh1/Nur1 and the cohibin components Lrs4/Csm1. Telomeric repeats are anchored to the nuclear periphery by binding to the INMP Mps3 or nucleoporin Nup170. The yKu70/80 heterodimeric complex combines with telomerase to bind to Mps3. Sir4 interacts with Mps3. Sir4 binds to Nup170 and forms a distinct complex with SUMO E3 ligase Siz2 and nuclear periphery protein Esc1. At *HM* loci, *HML* is linked to *HMR* via a long-range interaction. *HML* and *HMR* loci are clustered with the telomeres of chromosome III. *HMR* locus is anchored to nuclear periphery through Sir4-dependent manner. **B** Perinuclear anchoring of heterochromatin structural proteins in *S. pombe*. At fission yeast silent mating-type loci, the Rix1 complex (RIXC) binds to the silent chromatin domain and the boundary and nuclear rim protein Amo1 interacts with RIXC at the silent chromatin boundary for the anchoring of silent mating-type loci at the nuclear periphery. They construct concentrated foci of Swi6 and the FACT components Spt16 and Pob3 for heterochromatin maintenance and inheritance. Centromeric regions are anchored to the nuclear periphery through Lem2/Nur1. The nucleoporin components Npp106 and Nup211 lead to the nuclear periphery anchoring and silent chromatin formation of centromeres and telomeres

In *S. cerevisiae*, the positioning of heterochromatin structural proteins at the nuclear periphery is mediated by the interaction of heterochromatin structural proteins with nuclear membrane proteins and/or nucleoporins [10, 72–74]. At the rDNA loci of budding yeast, Lrs4/Csm1—two cohibin complex components—bind to Tof2 at Intergenic Spacer 1 (IGS1) and link rDNA loci to two perinuclear membrane proteins, Heh1 and Nur1 (Fig. 3A) [73]. Also, at the IGS1 of rDNA loci, Sir2 and Cdc14 is sequestered at the nucleolus by Net1 throughout interphase (G1/S/G2), thereby forming the RENT complex for silent chromatin formation [38, 75, 76] (Fig. 3A).

The anchoring of telomeres to the nuclear periphery is mediated by yKu70/80 heterodimer throughout the interphase and by SIR proteins, specifically Sir4, during the S-phase (Fig. 3A) [77, 78]. SUN domain protein Mps3, the nuclear periphery protein Esc1 (establishment of silent chromatin), and nucleoporin Nup170 are the innernuclear membrane proteins bound to telomeres (Fig. 3A) [74, 78–81]. Mps3 interacts with both Sir4 through its N-terminal acidic domain and yKu70/80 heterodimer [79, 81]. This interaction is important for perinuclear anchoring of telomeres and stability of telomeres [79, 81]. Nucleoporin Nup170 interacts with the chromatin remodeler RSC and Sir4, which stabilizes the binding of Sir4 to telomeric repeat regions and the perinuclear anchoring of telomeres [74, 80]. Moreover, Nup170 forms Sir4-associated Nup complex, composed of Nup170, Sir4, Esc1 and Siz2 (a SUMO E3 ligase) (Fig. 3A) [80]. This Sir4-associated Nup complex is distinct from holo-NPCs (nuclear pore complexes) and contributes to the organization of telomeres and anchoring of telomeres at the nuclear periphery [80].

In the silent mating loci of budding yeast, *HML* is linked to *HMR* via a long-range interaction [82]. *HML* and *HMR* loci are also clustered with the telomeres of chromosome III [82] (Fig. 3A). The anchoring of the *HMR* locus to nuclear periphery is dependent on Sir4 protein, but is not on yKu protein [83] (Fig. 3A). Instead, the yKu70/80 complex constraints the mobility of the *HML* locus [83] (Fig. 3A).

In *S. pombe*, heterochromatin regions are also anchored to the nuclear periphery by interaction with innernuclear membrane proteins and nucleoporins (Fig. 3A). A recent study identified that two nucleoporins, Npp106 and Nup211, led to the anchoring of centromeres and telomeres to the nuclear membrane (Fig. 3B) [10]. In addition, two inner nuclear membrane proteins, Lem2 and Nur1, localize centromeres and rDNA repeats at the nuclear periphery for the clustering of silent loci [10]. In silent mating-type loci of fission yeast, two inverted repeat elements, IR-L and IR-R, function as the boundary elements to sequester the H3K9me-Swi6 heterochromatin domain from the outer environment; these elements contribute to distinct heterochromatin domains by anchoring silent chromatin loci to the nuclear periphery [70, 72, 84]. A recent study by Holla et al*.* identified nuclear rim protein Amo1 as a perinuclear anchoring factor of silent mating loci in *S. pombe* [72]. To identify the heterochromatin factors that contribute to gene silencing at the *mat2P* locus within the silent mating loci of *S. pombe*, a deletion mutant of *REII* element, which showed weakened gene silencing at the *mat2P* locus, was crossed with a single-gene deletion library (Fig. 2B) [72]. A deletion mutant of *amo1* did not maintain gene silencing at the *mat2P* locus [72]. Amo1 was co-purified with two FACT components, Spt16 and Pob3, and the RNA processing factors, Rix1, and four Rix1-interacting components [72]. In the absence of Amo1, the H3K9me3 domain nucleates at the *cenH* region, but heterochromatin was not propagated nor maintained [72]. They concluded that local sequestration of the FACT complex prevents nucleosome turnover at silent mating-type loci, thereby enabling the maintenance of silent information [72]. In conclusion, in *S. cerevisiae* and *S. pombe*, heterochromatin loci are recruited to the nuclear periphery through interaction with inner nuclear membrane proteins and/or nucleoporins.

### Regional sequestration of heterochromatin structural proteins is critical for heterochromatin formation and gene silencing

A previous study by Andrulis et al*.* reported that artificial positioning of a gene into the nuclear periphery is sufficient to induce transcriptional silencing in *S. cerevisiae* [85]. At the study*, E* silencer at the silent *HMR* locus was replaced with multiple Gal4 binding sites to construct a “crippled silencer”, and this designed strain showed defects in *HM* silencing [85]. To artificially recruit this “crippled silencer” into the nuclear periphery, Andrulis et al. expressed a hybrid protein fusing Gal4 DNA-binding domain (GBD) and a nuclear membrane protein, Yif1 [85]. This hybrid protein could bind to both the “crippled silencer” and nuclear membrane, resulting in restoration of *HMR* silencing [85].

To confirm whether defective gene silencing is also restored by perinuclear positioning after concentrated foci of heterochromatin structural proteins at the nuclear periphery are collapsed, Taddei et al. performed similar experiments to those of Andrulis et al*.* [85] after deleting *yku70* [78]. In *yku70∆*, SIR complexes were dispersed from distinct subcompartments at the nuclear periphery to the entire nucleoplasm [78]. The *HMR* locus surrounded by “crippled silencers” was replaced by the *URA3* reporter gene and targeted to the nuclear periphery, but silencing was not restored [78]. These results suggest that perinuclear subcompartments with high concentration of heterochromatin structural proteins are required for heterochromatin formation and gene silencing.

Deletion mutants of Lrs4 or Csm1, the two condensin components linking rDNA repeats to nuclear periphery in *S. cerevisiae*, showed detachment of rDNA repeats from the nuclear periphery and nucleolus [73]. In addition, gene silencing was defective and unequal sister chromatid exchange (USCE) was increased at the mutant strains [73]. Thus, the nuclear anchoring of rDNA repeats through Tof2-Lrs4/Csm1 is important for stability of rDNA loci [73]. In the N-terminal acidic domain mutant of Mps3, the nuclear membrane protein bound to telomeres in *S. cerevisiae*, telomeric foci were dispersed from the nuclear periphery, and telomeric silencing was defective [79]. Knockout mutants of Npp106 or Nup211, the two nucleoporins for anchoring of centromeres and telomeres at nuclear periphery in *S. pombe*, showed defects in heterochromatin gene silencing [10]. Another study confirmed that a single deletion mutant of Lem2 and Nur1, the two innernuclear membrane proteins bound to centromeres and rDNA repeats in *S. pombe*, exhibited gene silencing defects at centromeres, telomeres, and silent mating-type loci [86]. These results imply that the positioning of heterochromatin loci to the nuclear periphery and formation of distinct nuclear subcompartments with high concentration of heterochromatin structural proteins is important for heterochromatin formation and gene silencing.

### Heterochromatin structural proteins are bound to nucleosomes with high affinity

We encountered a question of how concentrated foci of heterochromatin structural proteins contribute to heterochromatin structure and gene silencing. We will answer this question with the following arguments. Heterochromatin structural proteins are bound to nucleosomes with high affinity [13, 15]. In *S. pombe*, three domains of Swi6, namely CD, CSD, and the hinge region, contribute to the binding to nucleosome. In vitro peptide binding assay has shown that the CD of Swi6 binds to H3K9-methylated peptide with much higher specificity than unmodified peptide, but with weak affinity [87–89]. However, Swi6 has much higher affinity to nucleosomes than to peptides, suggesting the presence of alternative binding modes other than the binding between CD and methylated H3K9 [13]. It was reported that the hinge region of Swi6 can bind to nucleosomal DNA in a sequence-independent manner, and that CSD interacts with the globular region of histone H3 [90, 91]. Furthermore, the CSD of Swi6 binds to the φX(V/P)Xφ (φ, hydrophobic amino acid; X, any amino acid) motif in α1 helix of histone H2B, and is extensively bound to the nucleosome core [7].

The SIR complex in *S. cerevisiae* also binds to nucleosomes with high affinity [15]. Reconstituted nucleosome trimers, consisting of three nucleosomes, bind to the Sir2/3/4 holocomplex with high affinity (Kd = 10^−8^ M) [15]. The C-terminal of Sir3 interacts with the tail of histone H4, and both the C-terminal fragment of Sir3 and full-length Sir3 are capable of binding to unmethylated H3K79-containing peptide, suggesting the interaction between Sir3 and histone residues surrounding H3K79 [34, 92, 93]. Furthermore, the N-terminal bromo-adjacent homology (BAH) domain of Sir3 binds to histone H4 tail and nucleosomes [94]. Sir4 interacts with free DNA in a sequence-independent manner through its N-terminal region [15].

### Heterochromatin structural proteins are oligomerized for heterochromatin spreading

Heterochromatin structural proteins form oligomers [13, 14]. Swi6 forms dimer with another Swi6 via the CSD-CSD interface. The *K*_*d*_ value in solution is less than 17 nM, which suggests that Swi6 exists as a dimer at the nanomolar scale [13, 64]. Through mutational study, the Swi6-L315D substitution mutant with suppressed dimer formation was identified [64]. Swi6-L315D binds to H3K9-methylated peptides with similar affinity to Swi6 [13]. However, Swi6-L315D binds to mononucleosomes with significantly decreased affinity compared to Swi6; moreover, in the nucleosomal array context, the specific binding to H3K9-methylated nucleosomes is also significantly reduced [13]. Furthermore, in the Swi6-L315D mutant strain, microscopic analysis identified that distinct sequestered foci of Swi6 proteins disappeared, and that heterochromatic gene silencing was impaired [11].

In a previous study, an in vitro binding assay between reconstituted mononucleosomes and Swi6 revealed that the strength of the interaction is proportional to Swi6 concentration [13]. Specifically, the amount of mononucleosomes was fixed, whereas Swi6 was added gradually; nevertheless, the reaction did not reach saturation even though the concentration of Swi6 was high, which is suggestive of oligomerization at high Swi6 concentration [13]. A Swi6 dimer interacts with another dimer by the CD–CD (chromodomain) interface to form a tetramer [13]. In this tetramer, a remaining CD in each dimer binds to methylated H3K9 in a nucleosome, resulting in molecular bridges that link neighboring nucleosomes [13, 14]. Through mutational analysis, it was confirmed that the substitution of two residues of Swi6, V82E and Y131W, enhanced oligomerization [13]. Swi6-V82EY131W binds to H3K9-methylated nucleosomes with higher affinity than Swi6 [13]. In addition, gene silencing was enhanced at the endogenous *ura4* + reporter gene locus, and more Swi6 bound to the region in the Swi6-V82E Y131W substitution mutant [13]. In conclusion, Swi6 oligomerization is important for its affinity to nucleosomes, specificity to H3K9-methylated nucleosomes, and for the spreading of heterochromatin structure [13, 14].

Oligomerization is also important for the assembly of the SIR complex in *S. cerevisiae*. Previous studies revealed that in vitro, two SIR complexes bound to three nucleosomes in a nucleosomal array [15, 16]. To explain this stoichiometry, a model was designed, and it suggested that two SIR complexes are bound to linker DNA and interact with each other, collecting three nucleosomes [15, 16]. Sir4 forms a homodimer with Sir4 or a heterodimer with Sir3 through its coiled-coil domain at the C-terminal region [95, 96]. Also, Sir4 tightly binds to Sir2, thereby producing a Sir2/4 complex [33, 97].

A Sir3 dimer forms bridges between neighboring nucleosomes in an unmodified nucleosome array, resulting in a compact chromatin fiber [98]. Previous in vitro studies identified the multimer formation of Sir3 at high concentration [12]. Notably, under Sir3 overexpression, purified Sir3 from budding yeast could form multimers, including dimer, tetramer, and octamer, through self-association [12]. Sir3 immobilized on a surface plasmon resonance (SPR) chip binds to a purified Sir2/4 complex or histone H4 tail peptides with higher affinity when additional Sir3 was added [12]. This results suggest that Sir3 oligomerization enhances the binding of Sir3 to the Sir2/4 complex or histones [12]. In a Sir3-overexpressing strain, telomeric heterochromatin forms extended domains to distal regions from telomeres, and this extended domain consists of excess Sir3 relative to Sir2/4 [97, 99]. These results suggest that dose-dependent oligomerization of Sir3 exists in real yeast cells [97, 99]. Taken together, high concentrations of heterochromatin structural proteins promote oligomerization, which increases their binding affinity to nucleosomes and the spreading of heterochromatin structural proteins.

### Structures of heterochromatin structural proteins are changed by binding to nucleosomes

The conformation of both heterochromatin structural proteins, Swi6 in *S. pombe* and the SIR complex in *S. cerevisiae*, is changed while binding to nucleosomes. A study suggested that Swi6 is altered to the spreading-competent form by binding to methylated H3K9 [14]. In the CD of Swi6, there is an Alanine–Arginine–Lysine (ARK) loop, which is bound by CD [14]. Each of the two Swi6 dimers composing tetramer has one remaining CD and this CD occupies the CD of another Swi6 dimer, which forms the auto-inhibited conformation of Swi6 tetramer [14]. This auto-inhibited form of Swi6 tetramer could not link to neighboring nucleosomes [14]. Because H3K9 methylation competes with the ARK loop of a Swi6 dimer for binding to the CD of another Swi6 dimer, increasing H3K9 methylation hinders the auto-inhibited conformation of Swi6 tetramer, resulting in the open conformation of Swi6 tetramer, which contributes to the spreading of Swi6 [13, 14].

In *S. cerevisiae*, Sir2 is the NAD^+^-dependent histone deacetylase, which remove the acetyl group of the acetylated H4K16 residue [6, 100]. During the deacetylation process, O-acetyl-ADP-ribose (O-AADPR) is produced as a byproduct. The SIR complex is thought to bind to O-AADPR through an AAA^+^ motif in the C-terminal of Sir3 [12, 29]. The addition of O-AADPR increases the affinity of the Sir2/4 complex for Sir3 [12]. Electron microscope analysis showed that following the addition of O-AADPR, the conformation of the SIR complex is changed from the globular shape to the cylindrical shape, suggesting a conformational change in the SIR complex due to O-AADPR [12]. Furthermore, in vitro interaction assay revealed that the interaction between reconstituted nucleosome array with three nucleosomes and the SIR complex was increased by O-AADPR [15].

### Conformational changes in chromatin are triggered by the binding of heterochromatin structural proteins

A recent study identified that the structure of H3K9-methylated nucleosomes is changed by binding to Swi6 [7]. Notably, through structural analysis, it was confirmed that histone H3 and H4 residues buried in H3K9-methylated nucleosomes are exposed after interaction with Swi6 [7]. To restrict this conformational change, I62 residue of H3 and A33 residue of H4 were substituted with cysteine (H3I62C–H4A33C) [7]. The disulfide bond between two newly substituted cysteines represses the conformational change, causing nucleosomes to be maintained in the ground-state conformation despite binding with Swi6 [7]. The disulfide bond in histone H3–H4 reduces the inter-array self-association of nucleosomal array, which means that the interaction between methylated H3K9 and Swi6 induces conformational changes in nucleosomes and Swi6, leading to the assembly of heterochromatin structure [7].

In *S. cerevisiae*, the conformation of nucleosome structure is changed by the binding of the SIR complex or by the histone deacetylase activity of Sir2. A previous study performed analytical ultracentrifugation with nucleosome array assembled with recombinant histone proteins and DNA, and identified that formation of compacted chromatin fiber was suppressed by deletion of N-terminal stretch of histone H4 [101]. This result suggests that the tail in histone H4 is important for internucleosomal interaction and chromatin compaction [101]. Basic patches with five basic amino acids, KHRHK, exist in the middle of the H4 tail from the 16th to 20th. Through the structural study, the interaction between the N-terminal basic patch of histone H4 in a nucleosome with the acidic patch in H2A–H2B dimer of neighboring nucleosomes, which consists of glutamate and aspartate was identified [102]. H4K16 acetylation weakens the interaction between the H4 tail of one nucleosome and the acidic patch of neighboring nucleosome, thereby increasing the flexibility of H4 tail [103]. In addition, H4K16 acetylation strengthens the binding of H4 tail of one nucleosome to its own nucleosomal DNA, weakening the internucleosomal interaction [104]. The deacetylation of H4K16 by Sir2 antagonizes these processes and contributes to internucleosomal interaction and chromatin compaction. Moreover, the interaction between BAH domain at the N-terminal of Sir3 and nucleosomes induces conformational changes in the N-terminal tail of histone H4 that clamp H4 tail residues (H4R17, H4R19) to DNA to enhance silencing [105].

### Distinct physicochemical environment is formed in heterochromatin region in *S. pombe*

A study reported phase-separated liquid condensates in the presence of H3K9-methylated nucleosomes and Swi6 [7]. Phase-separated liquid condensates are considered the membrane-less organelle, which sequesters the inner environment from the outer environment [106]. When conformational changes in nucleosomes by binding to Swi6 were restricted by formation of the disulfide bridge between histone H3 and H4 residues (H3I62C–H4A33C), liquid condensate formation was suppressed [7]. Furthermore, in DimerX mutant (Swi6-L315D), which exhibited suppressed Swi6 dimer formation, liquid condensate generation was also inhibited [7]. However, LoopX mutant, which showed reduced oligomerization of Swi6, formed liquid droplets with H3K9-methylated nucleosomes, but the size was enlarged, suggesting that the surface tension and stability of the system were reduced [7].

Notably, the shape of liquid condensates was similar to nuclear subcompartments filled with Swi6 proteins in the nucleus [7]. Distinct Swi6 foci within the nucleus were collapsed in dimerX mutant, which did not produce liquid condensates in vitro [7, 11]. In LoopX mutant, which showed enlarged liquid condensates in vitro, Swi6 diffused at each foci in nucleus [7]. Thus, several mechanisms, including oligomerization and conformational changes in nucleosomes by binding to Swi6, are combined to form phase-separated liquid condensates in heterochromatin loci [7]. Moreover, this was considered a key mechanism for the formation of sequestered body of heterochromatin structural proteins at the nuclear periphery [7]. In *S. cerevisiae*, whether SIR complex foci at the nuclear periphery are also the phase-separated liquid condensates remains to be identified.

## Concluding remarks

Heterochromatin regions maintain a highly packaged chromatin structure throughout the cell cycle. Once heterochromatin is nucleated, heterochromatin is spread through the self-assembly of heterochromatin structural proteins, namely Swi6 in *S. pombe* and the SIR complex in *S. cerevisiae*, unless spreading is either prevented through heterochromatin boundaries or decreased gradually along the long transition zone in telomeres [1, 45].

In this manuscript, we discussed the sequestered nuclear subcompartments of heterochromatin structural proteins and their role in heterochromatin formation in *S. cerevisiae* and *S. pombe*. In addition to *S. cerevisiae* and *S. pombe*, regional sequestration of heterochromatin structural proteins and heterochromatin loci is also conserved in mammals. The concept that the human nucleus consists of two megabase scale compartments, A and B compartments, was firstly suggested through previous Hi-C experiments [107]. A compartment is transcriptionally active and more centrally located within the nucleus, but B compartment is transcriptionally repressed, gene-poor, and located at the nuclear periphery [107]. Moreover, the nuclear lamina, the protein network that lines the inner nuclear membrane, tethers heterochromatin loci into the nuclear periphery at the mouse system [108]. Integrated reporter gene was transcriptionally repressed by artificial positioning into the nuclear periphery in mouse fibroblast cells, which implies that positioning a gene locus into the nuclear periphery is associated with gene silencing in mammalian cells [109].

The HP1α of *Drosophila* and mammals, the homologs of *S. pombe* Swi6, also forms phase-separated liquid condensates in vitro and in vivo, highlighting the importance of sequestered nuclear subcompartments of heterochromatin structural proteins and heterochromatin regions [110, 111]. Similar to that by Swi6, phase-separated nuclear subcompartment by HP1α is induced by oligomerization, causing conformational changes in heterochromatin structural proteins. Notably, macromolecules interacting with HP1α were incorporated into the phase, whereas proteins non-interacting with HP1α were excluded from the phase [110, 111]. Thus, we suggest that regional sequestration of heterochromatin structural proteins and heterochromatin loci is crucial for heterochromatin formation in *S. cerevisiae* and *S. pombe,* and even in mammals.

## Data Availability

Not applicable.
